# Osteoporotic vertebral compression fracture accompanied with thoracolumbar fascial injury: risk factors and the association with residual pain after percutaneous vertebroplasty

**DOI:** 10.1186/s12891-022-05308-7

**Published:** 2022-04-11

**Authors:** Yang Luo, Tianyu Jiang, Hui Guo, Faqin Lv, Ying Hu, Lihai Zhang

**Affiliations:** 1 Department of Orthopedics, The First Medical Center of PLA General Hospital, No. 28 Fuxing Road, Beijing, 100853 P. R. China; 2 Department of Rehabilitation, The Second Medical Center of PLA General Hospital, Beijing, 100853 P. R. China; 3 Department of Ultrasound, The Third Medical Center of PLA General Hospital, Beijing, 100853 P. R. China; 4 Shenzhen Institute of Advanced Technology, Chinese Academy of Science, Shenzhen, 518055 P. R. China

**Keywords:** Osteoporotic vertebral compression fracture, compression fracture, percutaneous vertebroplasty, PVP residual pain, Thoracolumbar fascia

## Abstract

**Background:**

To explore the risk factors involved in the induction of thoracolumbar fascia (TLF) injury by osteoporotic vertebral compression fracture (OVCF), and the association between the residual pain after percutaneous vertebroplasty (PVP) and fascial injury.

**Methods:**

A total of 81 patients with single-segment OVCF, treated between January 2018 and January 2020 were included. The patients were grouped according to the existence of TLF injury. The patients’ general, clinical, and imaging data were accessed.

**Results:**

There were 47 patients in the TLF group and 34 in the non-injury group (NTLF group). In the TLF group, BMI (Body mass index) was significantly lower, while the prevalence of hypertension and sarcopenia were significantly higher (*P* < 0.05). The vertebral compression degree was higher, and the kyphosis angle of the injured vertebra was greater in the TLF group (*P* < 0.05). Cobb’s angle was not significantly different between groups. At 3-d after the operation, the VAS (Visual analogue scale) was 4.64 ± 1.78 and 3.00 ± 1.71, and the ODI (Oswestry disability index) was 67.44 ± 11.37% and 56.73 ± 10.59% in TLF and NTLF group, respectively (*P* < 0.05). However, at 3-m after the operation, the differences in the VAS score and the ODI between groups were not statistically significant. The area of fascial edema was not significantly associated with the pre- and post-operative VAS or ODI, but was positively correlated with the vertebral body compression degree (*R* = 0.582, *P* = 0. 029).

**Conclusion:**

Residual back pain after PVP is associated with TLF injury. Low BMI, hypertension and sarcopenia are risk factors of TLF injury, and sarcopenia may be the major factor.

## Introduction

Osteoporotic vertebral compression fracture (OVCF) is the most common fragility fracture that can result in severe lower back pain, disturbed sleep, kyphosis, decreased life quality, and increased mortality. It has been estimated that about 1.4 million patients are diagnosed with OVCF every year [1], which is the most common complication of osteoporosis [2]. As many as one-fourth of people older than 50 years old will experience at least one vertebral fracture during their lifetime [3], and one-third of the patients with compression fracture will suffer long-term pain [4]. Moreover, it has been estimated that just in the USA, almost 130,000 patients are hospitalized due to OVCF every year, and the annual direct medical expenditure for this disease amounts to about 10 to 22 billion dollars [5, 6].

Currently, percutaneous vertebroplasty (PVP) is the most commonly applied minimally invasive method for the treatment of vertebral compression fracture, which can substantially alleviate back pain in patients, allow them to conduct early ambulation, and prevent the complications, including hypostatic pneumonia and pressure ulcer induced by long-term immobilization [5–7]. Yet, some patients may still feel long-term lower back pain after PVP, which substantially affects daily activities. Some studies have demonstrated that the residual lower back pain after PVP is associated with infection, rib fracture, compression of the spinal cord or nerve root induced by bone cement leakage, and bone cement leakage related inflammatory responses [8–10].

Thoracolumbar fascia (TLF), also known as lumbodorsal fascia, is the deep fascia of the lumbar back that formed as the complex arrangement of multiple layers of fascia and aponeurosis. All the TLF layers merged at the lower back area to create a relatively thick fascia structure, which firmly attaches to the posterior superior iliac spine and sacrotuberous ligament. Such thoracolumbar composite (TLC) could help maintain the integrity of the lower lumbar vertebrae and sacroiliac joints, and functions like a bowstring during the anterior flexion of the spine [11, 12]. Magnetic resonance imaging (MRI) can show the co-existence of TLF injuries close to or far from the fractured vertebrae in some OVCF patients. The purpose of this article was to confirm the relationship between TLF injury and residual pain after PVP and to investigate the risk factors of TLF injury induced by OVCF.

## Methods

### Patients

A retrospective study of patients with single-segment OVCF treated between January 2018 and January 2020 was performed. Osteoporosis diagnosed by hounsfield unit (HU) measurement on computed topography (CT), which was L1 ≤ 110HU [13–15]. All patients are treated by the same team of doctors at a single hospital.

The patients’ general data including sex, age, height, body weight, BMI (body weight/height^2^), causes of the injury, hypertension, diabetes, and previous history of lumbar diseases, were collected. In addition, anteroposterior and lateral X-ray imaging of the spine, as well as 3-D CT and MRI imaging, were also conducted for all the patients.

Inclusion criteria were following: 1) > 50 years of age; 2) acute/ subacute fractures less than 6w; 3) with single-segment vertebral fracture of T6-L5; 4) the daily activities were substantially influenced by the back pain; 5) MRI of the vertebra showed low T1WI signal, high T2WI signal, and high signal of STIR sequence.

The exclusion criteria were following: 1) with multiple fractures or accompanied with organ injuries; 2) fractures caused by high-energy violence such as car accidents; 3) with spinal tumor or infection 4) with neural symptoms caused by encroachment/compression of the spinal canal from the fracture; 5) with kummell‘s disease; 6) could not tolerate the operation due to poor physical conditions.

### Diagnosis and measurement of TLF injury

**Fig. 1:**
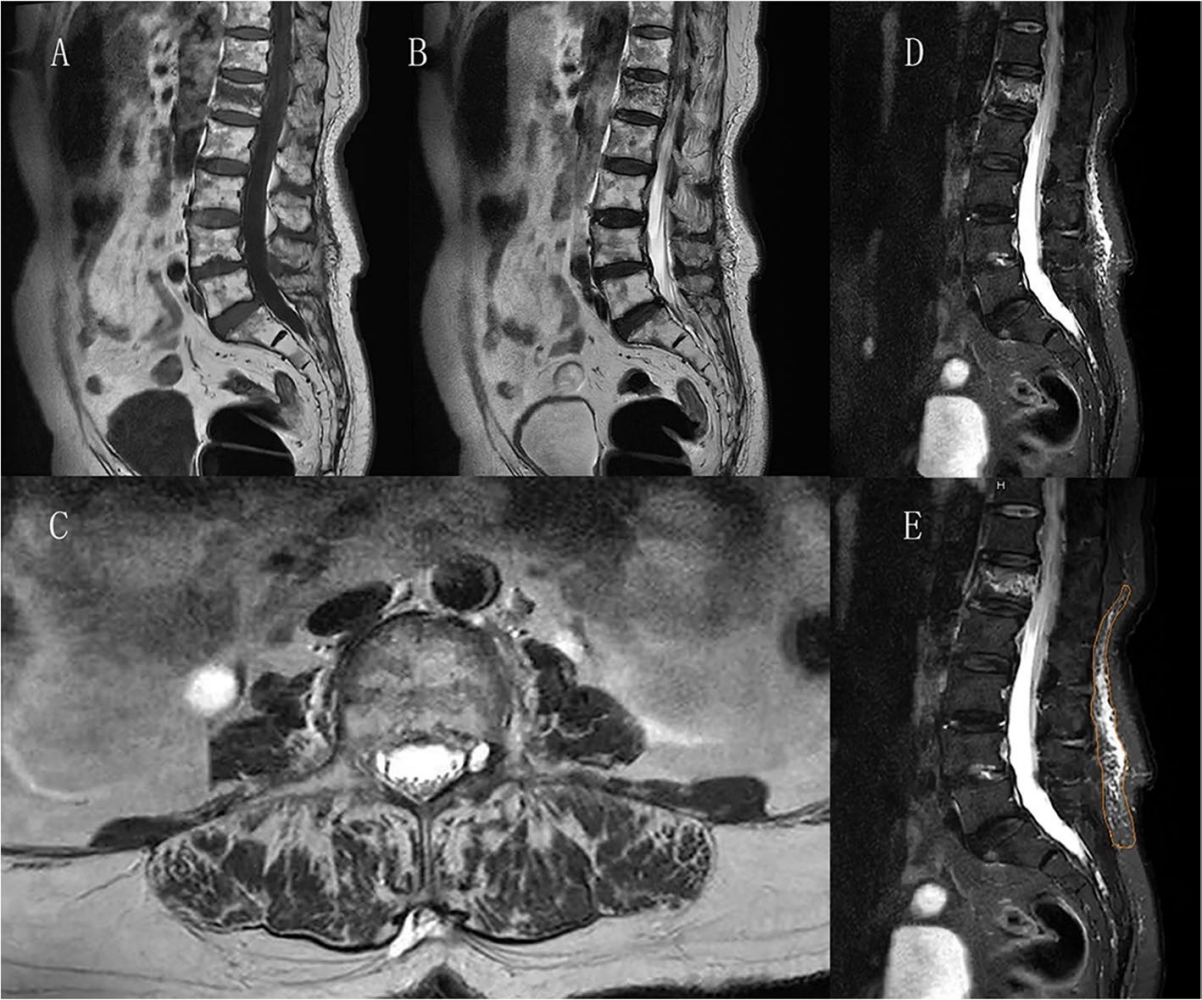
Vertebral compression fractures in the L1 vertebrae with TLF injury. **A** the T1WI image shows low signal intensity; **B** and **C**. T2WI image show high signal intensity; **D**. T2WI STIR sequence show high signal intensity; **E**. the site with the largest thoracolumbar fascial edema was selected

MRI examination was performed to diagnose TLF injury, which was defined as the signals of soft-tissue edema, namely low or iso T1WI signal, iso or high T2WI signal, and high signal of STIR sequence, on MRI images (Fig. 1A-D). The patients were divided into the TLF group or NTLF group, according to the existence of TLF injury.

Preoperative MRI examination was conducted to measure the area of fascial edema for the patients in the TLF group. Sagittal image of the STIR sequence was obtained, the site with the largest thoracolumbar fascial edema was selected (Fig. 1E), and the software of the imaging system was used to measure the edema area. The mean value of three measurements was calculated, and the result was reported as cm^2^.

### Diagnosis and measurement of sarcopenia

**Fig. 2:**
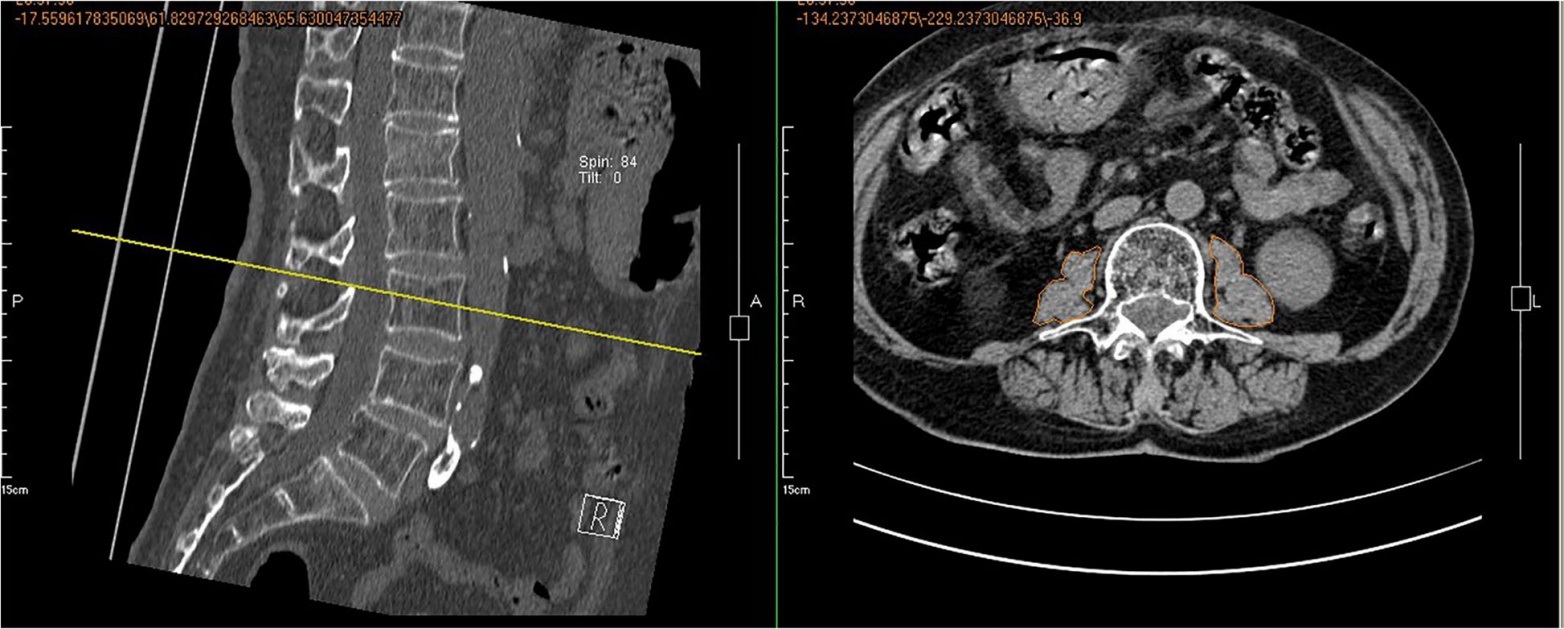
The TPA at level L3 was measured on CT by tracing the bilateral psoas major muscle outline

CT scanning was adopted to measure the total psoas area (TPA) of the patients in both groups [16, 17]. In brief, the cross-section of the CT image of the transverse processes of the third lumbar vertebra was reviewed, and the outlines of bilateral psoas major were manually sketched (Fig. 2). The software of the imaging system was used to measure the cross-section area, and the mean value of three measurements was calculated. TPA was calculated by dividing the cross-section area by the height squared and was described as mm^2^/m^2^. Sarcopenia was diagnosed if the TPA was < 385 mm^2^/m^2^ in females, or < 545 mm^2^/m^2^ in males [16].

### Measurement of vertebral fracture

**Fig. 3:**
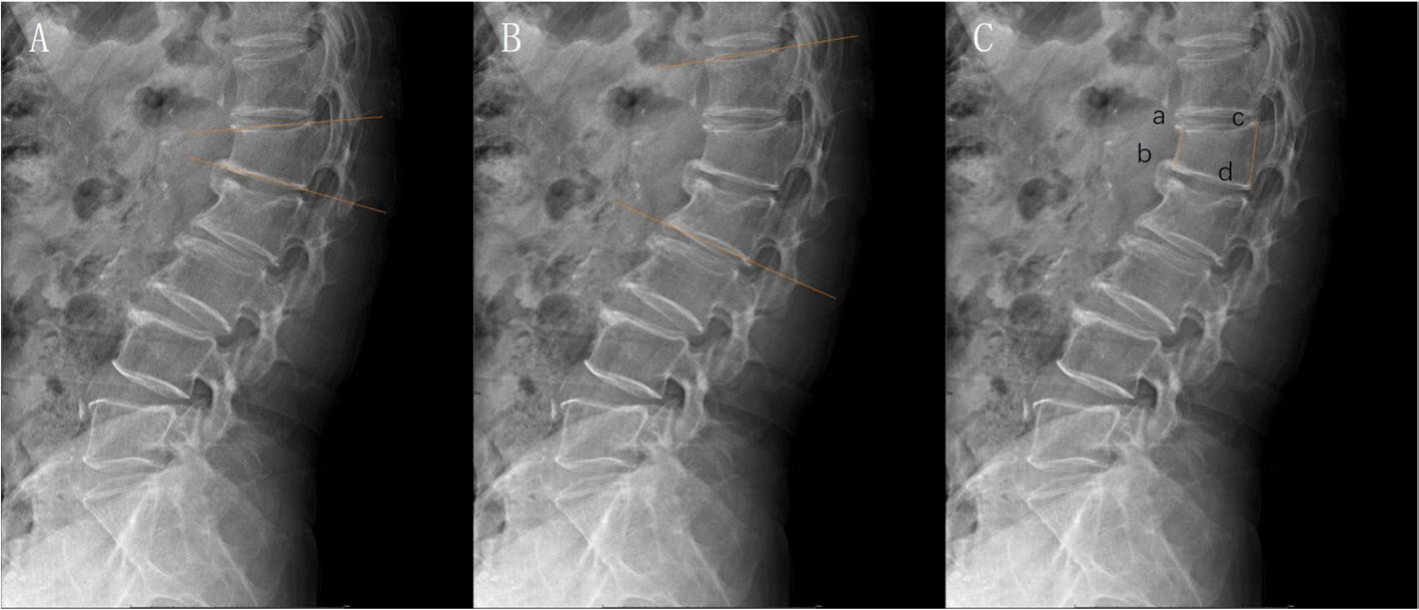
Preoperative X-ray imaging. **A** kyphosis angle; **B** Cobb’s angle; **C** vertebral compression degree (cd-ab/cd)

Preoperative X-ray imaging was conducted to measure the vertebral compression degree, Cobb’s angle, and kyphosis angle. In brief, the vertebral compression degree was calculated by (height of posterior vertebral margin – the height of the most severely compressed site) / height of posterior vertebral margin; the Cobb’s angle was calculated by measuring the angle between the line of the upper endplate of the superior vertebra and the line of the lower endplate of the inferior vertebra; for the measurement of kyphosis angle, a line was drawn between the anterior-superior horn and posterior-superior horn of the fractured vertebra, another line was drawn between the anterior-inferior horn and posterior-inferior horn of the fractured vertebra, and then the angle between the two lines was measured as the kyphosis angle (Fig. 3).

### Surgical methods

The patients were placed in a prone position with the abdomen dangling. The C-arm fluoroscopy system was used to visualize the anteroposterior image of the spine, and mark the position of the pedicle of the injured vertebra. After disinfection and draping, lidocaine was used for local infiltration anesthesia. Under the guidance of anteroposterior imaging through the fluoroscopy system, the puncture needles were inserted from the superolateral margins of the bilateral pedicles of the injured vertebra into the vertebral pedicle until reaching anterior 1/3 of the vertebra. The prepared bone cement was injected, and the X-ray imaging was continued to ensure there was no bone cement leakage. After the complete hardening of bone cement, the puncture needles were withdrawn. The patients rested in bed on the day of operation, and they were allowed to ambulate with orthosis on the day after the operation. Anti-osteoporosis drugs and non-steroidal anti-inflammatory drugs were routinely administered after the operation.

### Postoperative follow-up and measurement

X-ray imaging was conducted after the operation, and the rate of bone cement leakage after the operation was recorded.

Visual analogue scale (VAS) was used to assess the pain intensity, which could range from 0 to 10, with 0 indicating no pain at all, and 10 indicating drastic pain. The higher scores indicated more intense pain.

Oswestry disability index (ODI) was used to assess the degree of daily activity limitations from 10 items, including pain intensity, personal care, and walking. The scores for each item ranged from 0 to 5, with higher scores indicating more severe dysfunctions. The patients in this study were mainly elderly patients, the majority of whom had reduced sexual function, no social or tripping activities. Therefore, the ODI was modified in this study, and the total score was calculated according to the following equation:

$$total\ score= scores\ of\ the\ patients/\left(5\times the\ number\ of\ equations\ answered\right)\times 100\%$$

The VAS scores and ODI were recorded before, as well as at 3-d and 3-m after the operation. Meanwhile, we matched the demographics, vertebral compression degree, Cobb’s angle, and kyphosis angle of the TLF and NTLF group to reduce the influence of severity of OVCF on postoperative VAS and ODI scores, so as to more accurately evaluate the significance of TLF injury.

### Statistical analysis

SPSS22.0 software was used for the statistical analysis. Quantitative data were reported as means and standard divisions, and independent t-test was adopted for the comparisons between the two groups. The Fisher exact test compared qualitative data. The associations between the area of TLF edema and the influencing factors were assessed by the Spearman correlation test. *P* < 0.05 was considered statistically significant.

### Ethical statement

The study was approved by the Ethics Committee of the hospital.

## Results

### General characteristics of the patients

**Fig. 4:**
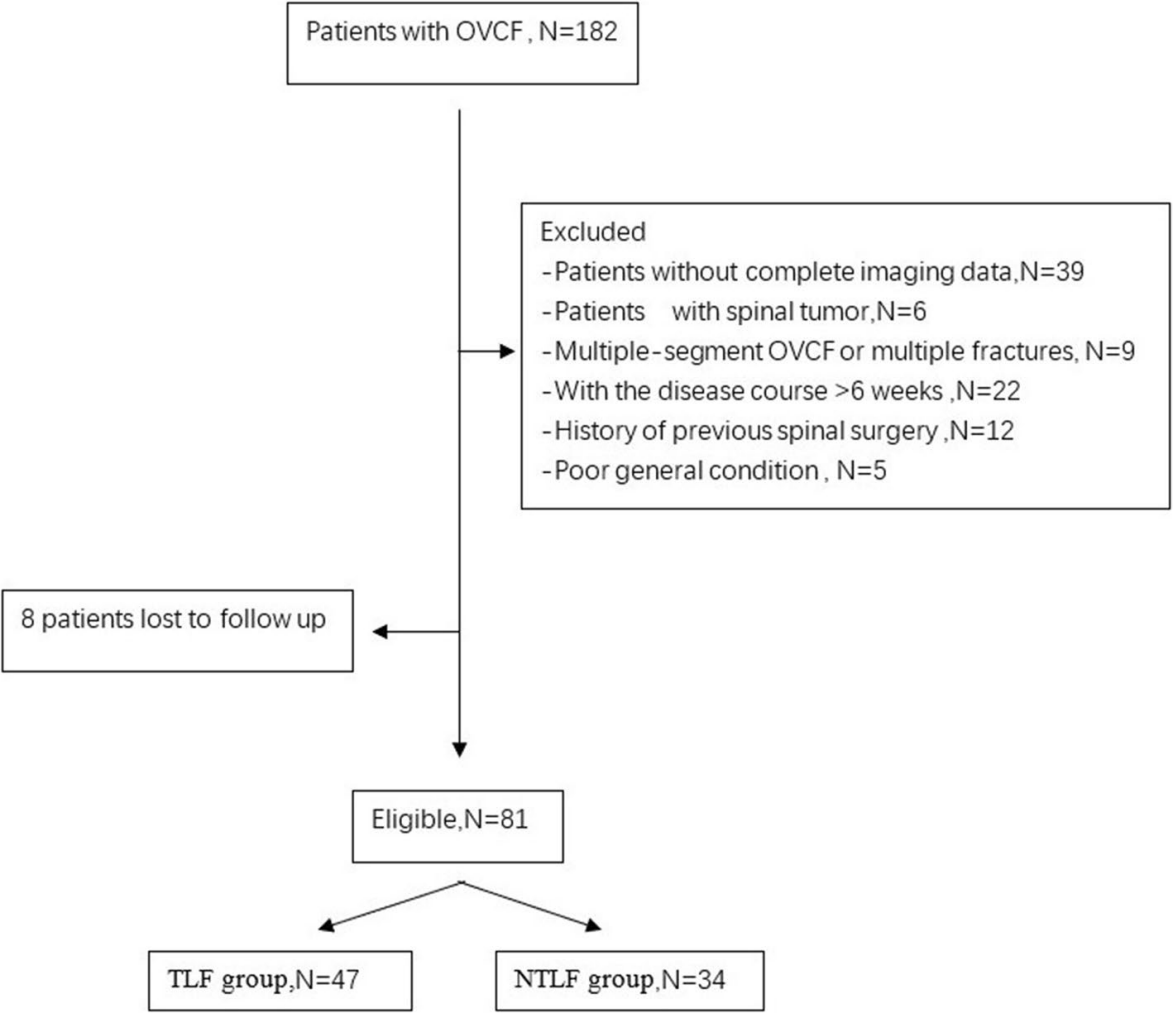
Flowchart describing the inclusion of patients

Finally, 81 patients, 24 males and 57 females, with the mean age of 73.9 ± 8.3 years (61–89 years), were included in this study (Fig. 4). Their vertebral fractures were as follows: 1 with T7 vertebral fracture, 8 with T11 vertebral fracture, 18 with T12 vertebral fracture, 36 with L1 vertebral fracture, 12 with L2 vertebral fracture, and 6 with L3 vertebral fracture. The fractures in 54 patients were caused by trauma such as falling, and 27 were caused by a mild external force such as cough and sneeze. All the patients were admitted to the hospital for back pain, and the time from injury to admission ranged from 6 h to 40 d.

Forty-seven patients were assigned to the TLF group and 34 to the NTLF group. The BMI was significantly lower in the TLF group compared to the NTLF group (22.61 ± 2.18 vs. 25.31 ± 3.16; *P* < 0.05), while the prevalence of hypertension was significantly higher in the TLF group compared to NTLF group (85.7% vs. 46.2%, *P* < 0.05). The other characteristics, including sex, age, and cause of injury, were not significantly different between the two groups (*P* > 0.05; Table 1).

**Table 1:** Baseline characteristics of patients

	TLF group (*n* = 47)	NTLF group (*n* = 34)	*P* value
Sex (Male/n)	17/47	8/34	0.224
Age (years)	76.00 ± 8.41	71.62 ± 7.86	0.175
Height(m)	1.63 ± 0.09	1.62 ± 0.09	0.779
Weight (kg)	59.9 ± 8.97	66.15 ± 10.45	0.108
BMI (kg/m2)	22.61 ± 2.18	25.31 ± 3.16	0.016*
Prevalence of hypertension(%)	27/47(57.4%)	10/34(29.4%)	0.012*
Prevalence of Diabetes(%)	7/47 (14.9%)	3/34(8.8%)	0.412
Prevalence of lumbar disorders(%)	10/47 (21.3%)	10/34 (29.4%)	0.402
Falls(%)	33/47 (70.2%)	21/34 (61.8%)	0.426
Time from injury to operation(%))	12.64 ± 9.57	17.85 ± 13.25	0.251

(*P < 0.05)

### Measurement of compression fracture

The vertebral body compression degree *w*as (45.43 ± 14.68)% and (34.86 ± 12.66)%, kyphosis angle of the injured vertebra was 13.89 ± 6.37 and 9.78 ± 4.00 in the TLF and NTLF groups, respectively; the observed differences were statistically significant (*P* < 0.05). The Cobb’s angle was 14.98 ± 10.45 and 8.69 ± 9.83 in the TLF and NTLF groups, respectively, which was not significantly different (*P* > 0.05). The results showed that the patients in the TLF group had more severe vertebral compression and higher kyphosis angle of the injured vertebra (Table 2).

**Table 2:** Comparison of degree of compression、Cobb’s angle and kyphosis angle

	TLF group (*n* = 47)	NTLF group (*n* = 34)	*P* value
degree of compression	45.43 ± 14.68	34.86 ± 12.66	0.025 *
Cobb’s angle	14.98 ± 10.45	8.69 ± 9.83	0.06
kyphosis angle	13.89 ± 6.37	9.78 ± 4.00	0.025*

(*P < 0.05)

### TPA

The TPA of the patients was measured as described in the previous reference [10]. Sarcopenia was diagnosed if the TPA was < 385 mm^2^/m^2^ in females, or < 545 mm^2^/m^2^ in males. Finally, 24 patients in the TLF group were diagnosed with sarcopenia, and the prevalence was 51.1%; 5 patient in the NTLF group was diagnosed with sarcopenia, and the prevalence was 14.7%. The prevalence of sarcopenia was higher in the TLF group compared to the NTLF group, the difference was statistically significant (*P* < 0.05). And the overall prevalence was 35.8%.

### Association with TLF edema area

The area of edema in the TLF group was (1.64–29.48) cm^2^. The associations between the edema area with vertebral body compression degree、kyphosis angle、Cobb’s angle、VAS score and ODI were assessed. The results showed that the edema area was not significantly associated with kyphosis angle、Cobb’s angle、pre- and post-operative VAS scores or ODI (*P* > 0.05), but was positively correlated with the vertebral body compression degree (*R* = 0.582, *P* = 0. 029).

### Operation findings

The operations were completed in all the patients with no severe complications, including pulmonary embolism, paraplegia, and perioperative deaths. Bone cement leakage was found in 27.7% (13/47) and 29.4% (10/34) patients by X-ray, respectively; however, the observed difference was not significantly different (*P* > 0.05). For the patients in the TLF group, the bone cement leaked to the intervertebral disc in 3 patient, along the paravertebral vein in 6 patients, and to the peri-vertebral soft tissues in 4 patient. For the patients in the NTLF group, the bone cement leaked to the intervertebral disc in 2 patient, along the paravertebral vein in 3 patient, and to the peri-vertebral soft tissues in 5 patients. No clinical symptoms were found in patients with bone cement leakage.

### Follow up and pain/function scoring of the patients

The preoperative VAS score and ODI were 9.14 ± 0.95 and 91.82 ± 5.67 in the TLF group, and 8.58 ± 1.78 and 84.08 ± 14.94 in the NTLF group, which were not significantly different between the two groups (*P* > 0.05). The VAS score and ODI in the two groups at 3-d and 3-m after the operation were both significantly different from the preoperative values (*P* < 0.05).

At 3-d after the operation, the VAS was 4.64 ± 1.78 and 3.00 ± 1.71, and ODI was 67.44 ± 11.37 and 56.73 ± 10.59 in the TLF and NTLF groups, respectively, both of which were significantly different (*P* < 0.05). At 3-m after the operation, the VAS was 2.14 ± 1.79 and 1.00 ± 0.95, and ODI was 30.04 ± 18.10 and 18.98 ± 10.94 in the TLF and NTLF groups, respectively; the observed differences were not statistically significant (*P* > 0.05) (Table 3).

**Table 3:** Comparison of VAS and ODI between the two groups

	TLF group (*n* = 47)	NTLF group (*n* = 34)	*P* value
preoperative VAS	9.14 ± 0.95	8.58 ± 1.78	0.344
preoperative ODI	91.82 ± 5.67	84.08 ± 14.94	0.113
VAS 3-d	4.64 ± 1.78	3.00 ± 1.71	0.025*
ODI 3-d	67.44 ± 11.37	56.73 ± 10.59	0.021*
VAS 3-m	2.14 ± 1.79	1.00 ± 0.95	0.051
ODI 3-m	30.04 ± 18.10	18.98 ± 10.94	0.077

*P < 0.05. VAS 3-d,VAS at 3-d after the operation; ODI 3-d,ODI at 3-d after the operation; VAS 3-m,VAS at 3-m after the operation; ODI 3-m,ODI at 3-m after the operation

After matching the demographics and severity of OVCF,42 patients in TLF group and 34 in NTLF group were included. At 3-d after the operation, the VAS was 4.46 ± 1.71 and 3.00 ± 1.71, and ODI was 67.05 ± 11.74 and 56.73 ± 10.59 in the TLF and NTLF groups, respectively, both of which were significantly different (*P* < 0.05). But there were no significantly different at 3-m after the operation (Table 4).

**Table 4:** Comparison of VAS and ODI between the two groups after matching the demographics and severity of OVCF

	TLF group (*n* = 42)	NTLF group (*n* = 34)	*P* value
Sex (Male/n)	13/42	8/34	0.472
Age (years)	75.62 ± 8.63	71.62 ± 7.86	0.228
degree of compression 44.25 ± 14.57		34.86 ± 12.66	0.092
Cobb’s angle	14.68 ± 10.81	8.69 ± 9.83	0.153
kyphosis angle	13.19 ± 6.05	9.78 ± 4.00	0.103
preoperative VAS	9.15 ± 0.99	8.58 ± 1.78	0.327
preoperative ODI	91.58 ± 5.83	84.08 ± 14.94	0.107
VAS 3-d	4.46 ± 1.71	3.00 ± 1.71	0.044*
ODI 3-d	67.05 ± 11.74	56.73 ± 10.59	0.031*
VAS 3-m	2.00 ± 1.78	1.00 ± 0.95	0.097
ODI 3-m	30.23 ± 18.82	18.98 ± 10.94	0.084

*P < 0.05. VAS 3-d,VAS at 3-d after the operation; ODI 3-d,ODI at 3-d after the operation; VAS 3-m,VAS at 3-m after the operation; ODI 3-m,ODI at 3-m after the operation

## Discussion

Yan et al [18] have divided OVCF patients into fascia injury group and non-fascia injury group, revealing that the residual pain after PVP was more severe in the fascia injury group than non-fascia injury group, and patients in the fascia injury group were older. However, they also included several patients with multi-segment OVCF or old fracture (the disease course could be as long as 6 months), which could potentially influence the findings. Also, the researchers did not investigate the risk factors of fascia injury, nor did they identify which patients were more likely to develop fascia injury. The purpose of this article was to confirm the relationship between TLF injury and residual pain after PVP and to investigate the risk factors of TLF injury induced by OVCF.

OVCF patients with single-segment vertebral fracture and the disease courses lasting for < 6 weeks, who were treated between January 2018 and January 2020, were included in this study. None of the patients were with multi-segment or old fractures, thus avoiding the potential influence of these factors on the results. The operations were successfully completed in all the patients, with no severe complications, including pulmonary embolism, paraplegia, or perioperative death. Bone cement leakage occurred in some patients in both groups, while no clinical symptoms were induced by the leakage.

The sex, age, height, body weight, cause of injury, time from injury to operation, the prevalence of diabetes, and prevalence of lumber diseases were not significantly different between the two groups. The BMI of the patients was 22.61 ± 2.18 and 25.31 ± 3.16 in the TLF and NTLF groups, respectively, which was significantly different (*P* = 0.016). The BMI in the TLF group was significantly lower, suggesting that the risk of TLF injury could be higher in patients with lower BMI. The prevalence of hypertension was 57.4 and 29.4% in the TLF and NTLF groups, respectively, and the hypertension prevalence was significantly higher in the TLF group compared to the NTLF group (*P* = 0.012).

We speculated that the lower BMI and higher prevalence of hypertension in the TLF group could be associated with sarcopenia, which is an aging-related progressive symptom of reduced systemic muscle mass and/or muscle strength or impairment of the physiological function of muscles [19, 20]. Sarcopenia and osteoporosis could be combined and termed as “movement disorder syndrome”, while fracture in the elderly could be considered as the consequence of sarcopenia and osteoporosis. Artiaco S et al. [21] found that about 30% of patients older than 50 years with distal radius fracture sufered by sarcopenia, and these patients usually suffer worse surgical outcomes. Male gender, aging, low BMI, elevated glycosylated hemoglobin, insufficient protein intake, and excessive total calorie intake are all risk factors of sarcopenia [22]. Previous studies have shown that hypertension prevalence is higher in patients with sarcopenia than in general people [23]. Park et al. [24] have studied the data of about 7000 patients and found that sarcopenia was one of the risk factors of hypertension, where the underlying mechanisms could be associated with insulin resistance [25] and inflammatory responses [26]. Therefore, we assumed that the higher hypertension prevalence in the TLF group could be associated with sarcopenia。.

To validate whether the prevalence of sarcopenia was different between the two groups, lumbar CT scanning was conducted in this study. Measuring TPA at the level of the transverse process of the third lumbar vertebra is a simple and rapid method for the assessment of sarcopenia [16, 17]. In this study, the prevalence of sarcopenia was evidently higher in the TLF group than in the NTLF group (51.1% vs. 14.7%; *P* < 0.05), which suggest that sarcopenia may be one of the risk factors for fascial injury. That result also explain the higher prevalence of hypertension in the TLF group. The causes of sarcopenia are multifactorial and can include disuse, altered endocrine function, chronic diseases, inflammation, insulin resistance, and nutritional deficiencies [20]. As mentioned above, male gender, aging, low BMI, elevated glycosylated hemoglobin, insufficient protein intake, and excessive total calorie intake are all risk factors of sarcopenia [22]. For this series of cases, low BMI may be one of the major factors contributing to the higher prevalence of sarcopenia in TLF group.

During the anterior flexion of the spine, the TLF could assist the lumbar back muscle in preventing over anterior flexion of spine, and thus protecting and stabilizing the lumbar vertebrae [11, 12]. However, over-flexion or excessive vertical force beyond the tolerance of TLF could induce fascial injury. Mentioned above, in the TLF group, there is a higher prevalence of hypertension which is one of the risk factors of falls. The patients with hypertension usually fell accompanied with symptoms of dizziness, who would lost the ability to control the back muscles in that kind of case. And The TLF is prone to damage due to the loss of the protection of the back muscles. Decreased muscle mass and strength in patients with sarcopenia will further increase the risk of fascial injury.

Compared with the NTLF group, the vertebral compression degree (*P* = 0.025) and kyphosis angle of the injured vertebra (*P* = 0.025) were both higher in the TLF group, which could be associated with the fact that for patients with more severe vertebral compression and higher kyphosis angle, the soft tissues of the lumbar back are exposed to higher stretch stress. Thus, the risk of fascial edema induced by the injury is also higher, which is more likely to occur when the back muscles strength are weakened in patients with sarcopenia. Yet, Cobb’s angle was not significantly different between the two groups (*P* = 0.06). We speculated that the Cobb’s angle would be between the lines of the endplate of the superior and inferior vertebrae of the injured vertebra, which also include the angle of the intervertebral space. Thus, the changes would be generally lower than the kyphosis angle. And the edema area was positively correlated with the vertebral body compression degree (*R* = 0.582, *P* = 0. 029), which means the more compressed the vertebra, the greater the edema area.

The VAS score and ODI at 3-d and 3-m after the operation in both groups significantly improved compared with the pre-operative values (*P* < 0.05), thus indicating that PVP effectively relieved the pain and improved the functions in the patients. The VAS score (*P* = 0.025) and ODI (*P* = 0.021) in the TLF group were both significantly higher than in the NTLF group at 3-d after the operation, thus suggesting that the improvements in pain and functions of patients with TLF injuries occurred more slowly, while the residual pain was more evident than in those without TLF injuries. At 3-m after the operation, the VAS score was 2.14 ± 1.79 and 1.00 ± 0.95 (*P* = 0.051), and ODI was 30.04 ± 18.10 and 18.98 ± 10.94 (*P* = 0.077) in the TLF and NTLF groups, respectively. Although the differences have no statistical significance, the findings showed the *P* values were both close to 0.05, and the VAS and ODI values in the TLF group were still evidently higher compared to the NTLF group. We speculated that these findings could be associated with the fact that after 3 months’ resting and physical therapy, the pain in the patients from the TLF group gradually alleviated, and therefore, the differences in the VAS and ODI values with the NTLF group gradually reduced. After matching the demographics, vertebral compression degree, Cobb’s angle, and kyphosis angle of the TLF and NTLF group, we got similar results which suggest that TLF injury is one of the major factors of residual back pain after PVP. The size of fascial edema area was not significantly associated with the pre- and post-operative VAS score or ODI, thus indicating that higher edema area was not necessarily accompanied by more intense pain.

The pain in patients from the TLF group was alleviated after PVP. However, the pain was still more intense than in NTLF group, which could be related to the following reasons: ①The injuries of the lumber back soft tissues, such as superficial fascia and muscles, could also lead to local pain; ②The branches of spinal nerve dorsal root travel in the TLF. The compression of nerve and stimulation of inflammatory factors after fascial injury and soft tissue edema could induce pain. PVP is only used to treat the vertebral fracture, but it may have no effect on pain induced by fascial and soft tissue injuries. Schilder et al have also investigated the mechanisms involved in the induction of pain by local edema after TLF injury in artificially induced TLF inflammation models, showing that after edema alleviated and inflammatory factors reduced, the pain also tended to gradually alleviate [27].

There are several limitations to the present study. First, residual pain after PVP is associated with several factors, while this study only investigated TLF injury. Second, in order to prevent the influence from various factors, strict eligible criteria were adopted in this study to select the patients, resulting in a relatively small sample size. Third,We also found fascia edema on MRI in some elderly patients with no history of trauma. We speculated that it was caused by long-term chronic stress damage to the TLF as the strength of the back muscles weakened in the aged. In the study, the history of lumbar disease was matched between the two groups to minimize the result bias. Fourth, we measure the area of fascia edema, not the volume. It is necessary to develop a new method of measuring volume to increase accuracy.

## Conclusion

In this study, we demonstrated the association between TLF injury and residual lower back pain after PVP and further investigated the risk factors of fascial injury. Low BMI, hypertension and sarcopenia are risk factors of TLF injury, and sarcopenia may be the major factor. A further prospective study involving a larger number of patients with long-term follow-up is necessary to confirm the results of our study,and the appropriate postoperative interventions also should be investgated to alleviate pain.

## Data Availability

The datasets used and/or analysed during the current study are available from the corresponding author on reasonable request.

## Abbreviations

TLF: Thoracolumbar fascia
OVCF: Osteoporotic vertebral compression fracture
PVP: Percutaneous vertebroplasty
TLC: Thoracolumbar composite
TPA: Total psoas area

## References

[1] Johnell O, Kanis JA (2006). An estimate of the worldwide prevalence and disability associated with osteoporotic fractures. Osteoporos Int.

[2] Rapado A (1996). General management of vertebral fractures. Bone.

[3] Jones G, White C, Nguyen T, Sambrook PN, Kelly PJ, Eisman JA (1996). Prevalent vertebral deformities: relationship to bone mineral density and spinal osteophytosis in elderly men and women. Osteoporos Int.

[4] Riggs BL, Melton LJ (1995). The worldwide problem of osteoporosis: insights afforded by epidemiology. Bone.

[5] Goz V, Errico TJ, Weinreb JH, Koehler SM, Hecht AC, Lafage V, Qureshi SA (2015). Vertebroplasty and kyphoplasty: national outcomes and trends in utilization from 2005 through 2010. Spine J.

[6] Rabei R, Patel K, Ginsburg M, Patel MV, Turba UC, Arslan B, Ahmed O (2019). Percutaneous vertebral augmentation for vertebral compression fractures: National Trends in the Medicare population (2005-2015). Spine.

[7] Balkarli H, Kilic M, Balkarli A, Erdogan M (2016). An evaluation of the functional and radiological results of percutaneous vertebroplasty versus conservative treatment for acute symptomatic osteoporotic spinal fractures. Injury.

[8] Mathis JM (2003). Percutaneous vertebroplasty: complication avoidance and technique optimization. AJNR Am J Neuroradiol.

[9] Lin CC, Shen WC, Lo YC, Liu YJ, Yu TC, Chen IH, Chung HW (2010). Recurrent pain after percutaneous vertebroplasty. AJR Am J Roentgenol.

[10] Hoffmann RT, Jakobs TF, Trumm C, Weber C, Glaser C, Reiser MF (2007). Vertebroplasty in the treatment of osteoporotic vertebral body fracture. Eur Radio.

[11] Willard FH, Vleeming A, Schuenke MD, Danneels L, Schleip R (2012). The thoracolumbar fascia: anatomy, function and clinical considerations. J Anat.

[12] Zhu Weikai SH, Yuanshan F, Shengbo Y, Chengming W, Hui S (2016). Development of anatomical structure of the thoracolumbar fascia. Chinese J Clinicalanatomy.

[13] Pickhardt PJ, Pooler BD, Lauder T, del Rio AM, Bruce RJ, Binkley N (2013). Opportunistic screening for osteoporosis using abdominal computed tomography scans obtained for other indications. Ann Intern Med.

[14] Hendrickson NR, Pickhardt PJ, Del Rio AM, Rosas HG, Anderson PA (2018). Bone mineral density T-scores derived from CT attenuation numbers (Hounsfield units): clinical utility and correlation with dual-energy X-ray absorptiometry. Iowa Orthop J.

[15] Zou D, Li W, Deng C, Du G, Xu N (2019). The use of CT Hounsfield unit values to identify the undiagnosed spinal osteoporosis in patients with lumbar degenerative diseases. Eur Spine J.

[16] Jones KI, Doleman B, Scott S, Lund JN, Williams JP (2015). Simple psoas cross-sectional area measurement is a quick and easy method to assess sarcopenia and predicts major surgical complications. Color Dis.

[17] Yamazaki Y, Kanaji S, Takiguchi G, Urakawa N, Hasegawa H, Yamamoto M (2020). Skeletal muscle loss after laparoscopic gastrectomy assessed by measuring the total psoas area. Surg Today..

[18] Yan Y, Xu R, Zou T (2015). Is thoracolumbar fascia injury the cause of residual back pain after percutaneous vertebroplasty? A prospective cohort study. Osteoporos Int.

[19] Cruz-Jentoft AJ, Baeyens JP, Bauer JM, Boirie Y, Cederholm T, Landi F, Martin FC, Michel JP, Rolland Y, Schneider SM, Topinková E, Vandewoude M, Zamboni M (2010). Sarcopenia: European consensus on definition and diagnosis: report of the European working group on sarcopenia in older people. Age Ageing.

[20] Fielding RA, Vellas B, Evans WJ, Bhasin S, Morley JE, Newman AB, Abellan van Kan G, Andrieu S, Bauer J, Breuille D, Cederholm T, Chandler J, De Meynard C, Donini L, Harris T, Kannt A, Keime Guibert F, Onder G, Papanicolaou D, Rolland Y, Rooks D, Sieber C, Souhami E, Verlaan S, Zamboni M (2011). Sarcopenia: an undiagnosed condition in older adults. Current consensus definition: prevalence, etiology, and consequences. International working group on sarcopenia. J Am Med Dir Assoc.

[21] Artiaco S, Fusini F, Pennacchio G, Colzani G, Battiston B, Bianchi P (2020). Sarcopenia in distal radius fractures: systematic review of the literature and current findings. Eur J Orthop Surg Traumatol.

[22] Qingheua H. Prevalence and risk factors of sarcopenia in middle-age and elderly patients with type 2 diabetes mellitus in Beijing.Dissertation. 2019. Peking Union Medical College.

[23] Han K, Park YM, Kwon HS, Ko SH, Lee SH, Yim HW, Lee WC, Park YG, Kim MK, Park YM (2014). Sarcopenia as a determinant of blood pressure in older Koreans: findings from the Korea National Health and Nutrition Examination Surveys (KNHANES) 2008–2010. PLoS One.

[24] Park SH, Park JH, Song PS, Kim DK, Kim KH, Seol SH, Kim HK, Jang HJ, Lee JG, Park HY, Park J, Shin KJ, Kim D, Moon YS (2013). Sarcopenic obesity as an independent risk factor of hypertension. J Am Soc Hypertens.

[25] Johannsen DL, Conley KE, Bajpeyi S, Punyanitya M, Gallagher D, Zhang Z, Covington J, Smith SR, Ravussin E (2012). Ectopic lipid accumulation and reduced glucose tolerance in elderly adults are accompanied by altered skeletal muscle mitochondrial activity. J Clin Endocrinol Metab.

[26] Mattace-Raso FU, Verwoert GC, Hofman A, Witteman JC (2010). Inflammation and incident-isolated systolic hypertension in older adults: the Rotterdam study. J Hypertens.

[27] Schilder A, Hoheisel U, Magerl W, Benrath J, Klein T, Treede RD (2014). Sensory findings after stimulation of the thoracolumbar fascia with hypertonic saline suggest its contribution to low back pain. Pain.

